# Intramuscular Olanzapine – a UK case series of early cases

**DOI:** 10.1186/1744-859X-6-11

**Published:** 2007-04-01

**Authors:** Chris J Bushe, Mark Taylor, Mathew Mathew

**Affiliations:** 1 Eli Lilly and Company Ltd, Basingstoke, UK; 2 Springpark Centre, Glasgow G22 5EU, UK; 3 Stonehouse Hospital, Dartford, DA2 6AU, UK

## Abstract

**Background:**

Clinical trials assessing efficacy and safety of Intramuscular (IM) Olanzapine in acute schizophrenia and acute mania have previously been undertaken in studies required for drug registration in patients who were required to give informed consent. These patients may have less severe forms of psychosis than patients treated in routine practice. Data derived from naturalistic practice following the launch of IM olanzapine may be helpful for clinicians in assessing efficacy and safety of IM olanzapine. The PANSS-EC scale used in the clinical studies may represent a tool that could be used in routine clinical practice.

**Case presentation:**

We report on an early unselected case series of 7 patients who received IM olanzapine in routine clinical practice settings in the UK. In this case series, olanzapine IM was generally effective, and no adverse events were reported. Adjunctive benzodiazepines were given concomitantly in 1 of the 7 subjects. This is relevant as concomitant benzodiazepines are not recommended for a minimum of 1 hour post IM olanzapine administration. PANSS-EC data was collected in 2 of the 7 subjects.

**Conclusion:**

Although patients had greater severity of psychosis than clinical trial patients there were no unexpected findings. In addition the PANSS-EC scale is a scale that may be useful in assessing the efficacy of IM antipsychotics in routine clinical practice.

## Background

During recent years more attention has been shown to rapid tranquilisation for the treatment of acute agitation in schizophrenia and bipolar disorder and the potential problems associated with it [1,2]. The majority of patients that require rapid tranquilisation have some form of serious psychosis and may also have physical problems such as biochemical disturbances and dehydration [3,4]

For many years the standard treatments used were droperidol and lorazepam [2]. Concerns over QTc prolongation led to cessation of use of droperidol in the UK [5] along with awareness that QTc prolongation could be more severe in patients administered intramuscular typical medications [1]. Droperidol usage is no longer permitted in the UK [5] and has been replaced in most instances by haloperidol. However, the use of intramuscular haloperidol is also well recognised to be associated with acute dystonic reactions and other extrapyramidal side effects [6,7].

Since Feb 2004 intramuscular olanzapine has been available in the UK and Europe and was the first licensed atypical available in Europe as a short acting intramuscular formulation. Clinical trials suggested low rates of occurrence for any QTc prolongation and acute dystonic reactions [1,7,8]. There were no differences found between placebo and IM olanzapine in terms of QTc prolongation from a pooled analysis of the IM olanzapine clinical trials.

Although IM olanzapine has been compared with placebo and haloperidol in acute agitation [7] associated with schizophrenia and with placebo and lorazepam in acute agitation [8] associated with mania, the studies required the patients to provide written informed consent to be included. Patients entered into these trials were thus likely to have had less severe forms of agitation than those who would be treated in routine practice. Furthermore the study of highly specific populations in randomised controlled trials (RCTs) (i.e. agitation associated with mania or schizophrenia) means that potential patients receiving the drug in clinical practice would have been excluded from RCTs for a variety of reasons including concomitant substance abuse. Thus patients treated in clinical practice are likely to be substantially different from those enrolled in RCTs. Preliminary clinical data derived from patients treated with IM olanzapine in a naturalistic setting may be of interest to clinicians.

The PANSS-EC scale is derived as a subscale of PANSS [9,10] and is a simple scale used to measure the degree of agitation. It formed the primary study endpoint in each of the main clinical IM olanzapine studies for registration [7,8] and may be a useful tool for clinicians in routine practice. The scale consists of 5 items (poor impulse control, tension, hostility, uncooperativeness, excitement) each being ranked from 1–7 giving a potential maximum score of 35 points.

We report on an early series of patients in whom intramuscular olanzapine was used and where in some cases the PANSS-EC scores were recorded as routine practice.

Psychiatrists treating patients in Psychiatric Intensive Care Units (PICU) and open wards in United Kingdom were asked to report data from their initial cohort of patients treated with IM olanzapine. The first 7 consecutive case reports received from any UK clinician have been included in this case series. These patients were treated in 2004–2005. Cases were reported informally to CB and were consecutively reported. No cases have been excluded. These patients represent an unselected group of patients from units around the UK whom were treated with IM olanzapine at a time when the predominant IM medications being prescribed were haloperidol and lorazepam.

Patients provided retrospective informed consent to the anonymised use of their details. The data is reported in a descriptive manner and no formal statistical analyses have been performed. In those patients where PANSS-EC scores have been measured this formed part of the routine assessment of patients within that unit at the time.

## Case presentations

### Case 1

A 40-year-old woman with a diagnosis of bipolar illness presented in an acute manic state. Previous but not current medication included depot clopixol and lithium. There was recent usage of heroin and other illicit drugs. Current dosage of diazepam was 80 mg/day. After admission 2 doses of clopixol acuphase (75 and 100 mg respectively) given over 5 days had little effect other than some sedation. Immediately prior to being given IM olanzapine 10 mg her behaviour was loud, over familiar and intrusive. The patient requested an IM sedative and was not detained under the Mental Health Act (MHA). Neither concomitant benzodiazepines nor anticholinergics were given. PANSS-EC scores shown in table 1. The nursing staff reported the patient as being much "quieter" but not asleep after 60 minutes. Following a single dose of intramuscular olanzapine, depot clopixol was reinstated with valproate. No adverse events were reported.

**Table 1:** PANSS-EC scores of Case 1

Time (minutes)	0	30	60	120
PANSS-EC	24	8	8	6

### Case 2

A 28-year-old woman with a diagnosis of schizophrenia presented in a psychotic and uncooperative state refusing medication. Medication prior to admission was 800 mg quetiapine and 1 mg lorazepam daily. Her behaviour became overtly aggressive including making a hole in the hospital bedroom wall and attempted assaults on staff. She was placed under the MHA and given IM olanzapine 10 mg. Neither concomitant benzodiazepines nor anticholinergics were given. PANSS-EC scores shown in table 2.

**Table 2:** PANSS-EC scores of Case 2

Time (minutes)	0	30	60	120
PANSS-EC	25	22	12	4

Nursing observations showed that the patient was asleep after 120 minutes post-IM (01.00) and remained "quieter and more relaxed for the next 2 days". Patient feedback was that she felt calmer after IM olanzapine. No adverse events were reported by the patient or physician. Patient however continued to refuse oral medications and currently receives depot risperidone.

### Case 3

A 49-year-old woman with a long standing history of bipolar affective disorder presented in a mixed affective psychotic state having had no relapses for 2 years. Her previous medication regimens included citalopram, lithium, valproate and lamotrigine. At admission her medication was lamotrigine 100 mg. Her behaviour was reported as "aggressive, confrontational and entertaining beliefs suggestive of delusional jealousy and suspiciousness about family".

Oral medication was refused and the patient was sectioned under the MHA. IM olanzapine 10 mg was given and no concomitant benzodiazepines or anticholinergics were administered. Nursing staff reported a moderate degree of tranquilisation (calm, relaxed and not sleeping) and that confrontation was avoided. Little effect upon delusional beliefs was noted. The patient began to engage with staff within 36 hours post IM olanzapine and at which stage oral olanzapine was accepted. No adverse events were reported.

### Case 4

An 18-year old male with a diagnosis of schizophrenia was admitted in an acutely psychotic state under the MHA. He refused oral medication and presented in an extremely agitated state expressing paranoid delusions such as "people have put fish bones in my food". He was not currently on medication (although previously had received olanzapine 10 mg orally) and was known to have used illegal substances in the past. There was an additional forensic history. He was treated with 10 mg olanzapine IM and the nursing notes state, "he soon went to sleep". Around 12 hours later he became agitated again and barricaded himself in his room. He was given 10 mg olanzapine IM and 2 mg IM lorazepam. The nursing notes report that within 30 minutes he was "settled" and had "calmed down". Around 2 hours post-injection he was asleep. Subsequently he was transferred onto olanzapine 20 mg orally. No adverse events were reported.

### Case 5

A 65-year old male with a diagnosis of schizoaffective disorder was admitted under the MHA in an extremely agitated state and expressing suicidal ideation. There was evidence of persecutory delusions. Around midnight he received 10 mg olanzapine IM. No benzodiazepines were administered. The nursing notes report that he "slept well" and "settled" soon after medication. No concomitant benzodiazepines were given. No adverse events were reported.

### Case 6

A 40-year old female with a diagnosis of bipolar disorder was admitted in an acutely manic state. Her normal medications included olanzapine 20 mg and sodium valproate. The nursing staff report that she was "restless, agitated, verbally aggressive and had evidence of an elevated mood". She was treated with 10 mg olanzapine IM. The notes report that she settled after medication and "slept undisturbed". No benzodiazepines were administered. Within the first 24 hours after IM olanzapine she was commenced on depot clopixol 200 mg/weekly. No adverse events were reported.

### Case 7

A 46-year old male with a 10-year history of schizophrenia was admitted in an acutely psychotic state. He had relapsed having been changed from IM depot haloperidol to risperidone LAIM. For the 2 weeks prior to admission he had received additional oral risperidone and sodium valproate He had showed features of paranoid and grandiose delusions together with elevated mood and had been asked to leave work, as "colleagues could not understand his talk". On admission, he was given a single injection of IM olanzapine 10 mg at around 18.00. No concomitant benzodiazepines were administered. The nursing reports state that "later that night he was no longer laughing without reason and was quiet. He slept well". The response was not maintained and he was reported as grossly psychotic again at midday the next day. No adverse events were reported.

## Discussion

In general terms and acknowledging the limitation of reporting only a small number of cases, our findings are not dissimilar from the clinical trials. Responder rates in the IM olanzapine RCTs at 2 hours post IM administration were 63–80% in schizophrenia patients and 81% in bipolar subjects [11]. In this review of 7 cases, the majority of the patients received only a single IM olanzapine injection without need for concomitant benzodiazepines or anticholinergics as in the clinical trials, where these medications were commonly prescribed. In 5 of the 7 cases, resolution of the behavioural symptoms was reported soon after the initial treatment with olanzapine IM, and in 2 of those cases, a response was reported within an hour. In 4 of the 7 cases patients were either acutely manic or were reported as having schizoaffective symptomatology with prominent mood symptoms. Only 1 of the 7 patients needed more than one injection, which could be consistent with the data from the mania RCT in which 26% patients needed more than one olanzapine injection [8]. No spontaneous adverse events were reported in this small naturalistic series.

The use of IM olanzapine as reported in this small number of cases has not led to any unexpected findings. The PANSS-EC scale is a quick and fairly routine scale to be able to use in everyday clinical practice and usage has been adopted by some UK clinicians. The falls seen in PANSS-EC in this cohort however are individually greater than those measured in the registration clinical trials. The decreases in PANSS-EC at 120 minutes in our 2 patients were 21 and 18 points respectively, which compares to a mean decrease of 6 points in the clinical trials at the same time point [7,8] There are a number of possible explanations for this, which include better awareness of the PANSS-EC scale by trial investigators with more accurate measurements, or that greater decreases may be seen in the naturalistic population studied who commence with higher PANSS-EC scores. The mean PANSS-EC entry scores in both the schizophrenia and mania IM agitation studies were around 18 [7,8] This contrasts with 24.5 (range 24–25) in this small series. The difference in entry PANSS-EC is not unexpected as the requirement for informed consent in the clinical trials precludes enrolling particularly hostile or oppositional individuals (as seen in this naturalistic case series), which in turn would lead to lower PANSS-EC scores. However even in this series not all patients were detained under the MHA and in one case even requested IM olanzapine treatment.

The use of benzodiazepines as either a primary or concomitant treatment is widely debated. The product license for IM olanzapine in the UK requires that no patient should receive benzodiazepines for at least 1 hour after IM injection. Furthermore the product license states that when benzodiazepines are being taken prior to admission to hospital caution should be exercised when administering IM Olanzapine and appropriate clinical monitoring should be undertaken in accordance with the olanzapine product license. In this small group 1/7 patients had been taking benzodiazepines immediately prior to admission, and also in 1 other cases adjunctive benzodiazepines were subsequently used. In this case, IM lorazepam 2 mg was given at the same time as IM olanzapine and thus not in line with the license which recommends not giving adjunctive benzodiazepines for a minimum of 1 hour post IM olanzapine injection. However in this individual case, no adverse events were observed and the patient received lorazepam after being given a second dose of IM olanzapine having responded partially for a relatively short period of time following the first dose.

The responder rates measured in the trials of IM olanzapine and comparator treatments suggest that good rates of response will be seen with only a single injection of any active treatment (olanzapine, lorazepam, and haloperidol). Similarly we observed in our series that good responses were seen in moderately severely agitated patients given mainly a single 10 mg injection of IM olanzapine. In our small series 6 patients responded well to a single IM olanzapine injection. The response in the other patient given 2 injections of IM olanzapine was poor initially with a short term response being maintained only temporarily necessitating an additional injection.

Although data was being in some cases collected routinely to assess PANSS-EC there was however no formal charting collecting parameters like blood pressure and other vital signs. With usage of IM olanzapine it is imperative that clinicians adhere to recommendations made in the product information with regard not only to dosages but also safety monitoring.

The nursing observations and patient feedback in this study were similar to the clinical trial observations that IM olanzapine was associated with a calming effect with rapid onset of action but with no evidence of over sedation. Some patients are reported to have slept following dosing but the medication was administered during the night.

Nothing inconsistent with the data from the clinical trials was observed despite there being a more severe degree of agitation in our naturalistic population in at least some patients as observed from the cases in which PANSS-EC data was reported.

## Key points

• The PANSS-EC scale is an easy to administer and easy to record scale that might find use in the wider clinical setting. The simplicity of the scale suggests that it could have routine usage in PICU's and other wards where any intramuscular treatments for patients with acute psychosis are used.

• Clinical monitoring as suggested within the olanzapine product license to include cardiovascular and respiratory parameters should also be routinely recorded.

## Limitations

There are clear limitations to any interpretations of data deriving from this small number of early case reports and bias in the collection and reporting of the data cannot be excluded.

## Competing interests

Chris Bushe is an employee of Eli-Lilly UK.

Dr M Taylor has received hospitality from various pharmaceutical companies and has received fees for lecturing for Eli Lilly UK

## Authors' contributions

CB instigated the data collection and authored the initial manuscript. MT and MM acquired the data and provided further authorship input.

## References

[B1] Lindborg S, Beasley C, Alaka K, Taylor C (2003). Effects of intramuscular olanzapine versus haloperidol and placebo on QTc intervals in acutely agitated patients. Psychiatry Research.

[B2] Jones B, Taylor C, Meehan K (2001). The efficacy of a rapid acting formulation of intramuscular olanzapine for positive symptoms. J Clin Psychiatry.

[B3] Battaglia J, Lindborg S, Alaka K, Meehan K, Wright P (2003). Calming versus sedative effects of intramuscular olanzapine in agitated patients. Am J Emerg Med.

[B4] Tesar G, Stern T (1986). Evaluation and treatment of agitation in the intensive care unit. J Intens Care Med.

[B5] Anonymous (2001). QT prolongation with antipsychotics. Current problems in pharmacovigilance.

[B6] Glazer WM (2000). Extrapyramidal side effects, tardive dyskinesia and the concept of atypicality. J Clin Psychiatry.

[B7] Wright P, Birkett M, David SR, Meehan K, Ferchland I, Alaka KJ, Saunders JC, Krueger J, Bradley P, San L, Bernardo M, Reinstein M, Breier A (2001). Double blind placebo-controlled comparison of intramuscular olanzapine and intramuscular haloperidol in the treatment of acute agitation in schizophrenia. Am J Psychiatry.

[B8] Meehan K, Zhang F, David S, Tohen M, Janicak P, Small J, Koch M, Rizk R, Walker D, Tran P, Brier A (2001). A double blind randomised comparison of the efficacy and safety of intramuscular injections of olanzapine, lorazepam or placebo in treating acutely agitated patients diagnosed with bipolar mania. J Clin Psychopharmacology.

[B9] Kay SR, Sevy S (1990). Pyramidical model of schizophrenia. Schizophrenia Bulletin.

[B10] Kay SR, Fiszbein A, Opler LA (1987). The positive and negative syndrome scale (PANSS) for schizophrenia. Schiz Bull.

[B11] Wagstaff A, Easton J, Scott L (2005). Intramuscular Olanzapine – a review of its use in the management of acute agitation. CNS Drugs.

